# Analysis of Lipid Metabolism, Immune Function, and Neurobehavior in Adult C57BL/6JxFVB Mice After Developmental Exposure to di (2-ethylhexyl) Phthalate

**DOI:** 10.3389/fendo.2018.00684

**Published:** 2018-11-21

**Authors:** Liana Bastos Sales, Joantine C. J. van Esterik, Hennie M. Hodemaekers, Marja H. Lamoree, Timo Hamers, Leo T. M. van der Ven, Juliette Legler

**Affiliations:** ^1^Department of Environment and Health, Vrije Universiteit Amsterdam, Amsterdam, Netherlands; ^2^Department of Pathobiology, Faculty of Veterinary Medicine, Utrecht University, Utrecht, Netherlands; ^3^Center for Health Protection, National Institute for Public Health and the Environment (RIVM), Bilthoven, Netherlands; ^4^Institute for Risk Assessment Sciences (IRAS), Utrecht University, Utrecht, Netherlands; ^5^Utrecht Institute for Pharmaceutical Sciences, Utrecht University, Utrecht, Netherlands

**Keywords:** early-life exposure, DEHP, lipid metabolism, neurobehavior, immunofunction, sex-specificity, mouse model, developmental exposure and adult disease

## Abstract

**Background:** Developmental exposure to di (2-ethylhexyl) phthalate (DEHP) has been implicated in the onset of metabolic syndrome later in life. Alterations in neurobehavior and immune functions are also affected by phthalate exposure and may be linked to the metabolic changes caused by developmental exposure to DEHP.

**Objectives:** Our goal was to study the effects of developmental exposure to DEHP in the context of metabolic syndrome by integrating different parameters to assess metabolic, neurobehavioral, and immune functions in one model.

**Methods:** Female C57BL/6J mice were exposed to DEHP through the diet during gestation and lactation at doses ranging from 3.3 to 100,000 μg/kg body weight/day (μkd). During a 1-year follow-up period, a wide set of metabolic parameters was assessed in the F1 offspring, including weekly body weight measurements, food consumption, physical activity, glucose homeostasis, serum lipids, and endocrine profile. In addition, neurobehavioral and immune functions were assessed by sweet preference test, object recognition test, acute phase protein, and cytokines production. Animals were challenged with a high fat diet (HFD) in the last 9 weeks of the study.

**Results:** Increased free fatty acids (FFA) and, high density lipoprotein (HDL-C) were observed in serum, together with a decrease in glycated hemoglobin levels in blood of 1-year old male DEHP-exposed offspring after HFD challenge. For the most sensitive endpoint measured (FFA), a lower bound of the 90%-confidence interval for benchmark dose (BMD) at a critical effect size of 5% (BMDL) of 2,160 μkd was calculated. No persistent changes in body weight or fat mass were observed. At 33,000 μkd altered performance was found in the object recognition test in males and changes in interferon (IFN)γ production were observed in females.

**Conclusions:** Developmental exposure to DEHP combined with HFD in adulthood led to changes in lipid metabolism and neurobehavior in male offspring and cytokine production in female offspring. Our findings contribute to the evidence that DEHP is a developmental dyslipidemic chemical, however, more research is needed to further characterize adverse health outcomes and the mechanisms of action associated with the observed sex-specific effects.

## Introduction

Metabolic syndrome (MetS) is a pathologic condition characterized by abdominal obesity, insulin resistance, hypertension, dyslipidemia, and hyperglycemia (1). The MetS prevalence among adults in the US (24–34%) (2) and in China (24.5%) (3) indicates its epidemic proportions. Many interventions have targeted excessive food intake and sedentary lifestyle without success, indicating the need for a better understanding of the factors involved in the pathogenesis of MetS (4). Increasing attention has been given to the developmental origins of health and disease (DOHaD) hypothesis (5), in which early exposure to stressors during critical periods of life may induce effects that manifest later in life. Exposure to environmental chemicals is one of the stressors which has been linked to the high MetS prevalence rates, and evidence is growing that exposure during periods when adipocytes are differentiating and/or organs as pancreas, liver, and brain are developing can lead to disruption of normal development and alterations in the homeostatic control of adipogenesis and energy balance (6).

Di(2-ethylhexyl) phthalate (DEHP) is an example of an environmental chemical which has been linked to metabolic disorders. It is used to add flexibility to polyvinyl chloride polymers (7). Human exposure occurs mainly orally, by migration of the chemical from the packaging to food such as fatty fish, meat and milk (8). DEHP metabolites have been detected in human samples of blood and urine, confirming the ubiquitous presence of DEHP (8, 9). DEHP is an endocrine disrupting chemical (EDC) and has putative obesogenic properties as reported in epidemiological, animal and *in vitro* studies (10). The first line of evidence that developmental exposure to DEHP may promote metabolic disorders was the increases in serum cholesterol (CHOL), triglycerides (TGs) and glucose reported in multiple studies (11–13). Recently, Wassenaar and Legler (14) conducted a systematic review and meta-analysis of experimental rodent studies with DEHP, and reported a statistically significant positive association between developmental exposure to DEHP and increased fat pad weight. However, further associations with triglycerides, free fatty acids (FFA) and leptin could not be analyzed due to few or no data available. The second line of evidence for a role for DEHP in metabolic disorder is its effects on neurobehavior, given the interaction of the brain with other key metabolic organs via signaling molecules and neuronal connections at the basis of pathways such as regulation of appetite (15). Schmidt et al. (16) reported an alteration in food intake in female C3H/N mice after an 8-week exposure to DEHP, and Barakat et al. (17) reported an impairment in neurobehavior and recognition memory in male CD-1 mice after prenatal exposure to DEHP. In addition, inflammation and activation of the immune system have been observed in abdominal obesity and may have a role in the pathogenesis of obesity-related metabolic disorders (18). Specifically, *in utero* exposure to DEHP increased serum levels of C-reactive protein (CRP) and tumor necrosis factor (TNF), increased TNF levels in adipose tissue homogenates, and promoted a focal macrophage infiltration in whole-adipose tissue, suggesting a systemic and local adipose inflammation in the adult male offspring of Sprague-Dawley rats (19).

Given the previous findings in separate studies and the need to have a global view of the effects of developmental exposure to DEHP, we hypothesized that early exposure to DEHP may affect metabolic, neurobehavioral, and immunological domains in an integrated manner. Our aim was to perform a combined assessment of metabolism disruptive properties, neurobehavior, and immune function following developmental exposure to DEHP. To achieve this, metabolic alterations in adult mouse offspring were studied after maternal dietary exposure during gestation and lactation, using seven doses ranging from doses relevant to human diet exposure (20) to a dose approximating the no observed adverse effect level (NOAEL) for developmental toxicity in CD-1 mice (21). The offspring was followed for one year and parameters related to energy balance (weekly body weight measurements, food consumption, and physical activity) and metabolism (glucose homeostasis, serum lipids, and endocrine profile) were assessed. In the last 9 weeks of the follow-up, offspring was challenged with a high fat diet (HFD) to test potential disturbances in their metabolic homeostatic capacity. A sweet preference test and an object recognition test were performed to assess neurobehavior whereas levels of CRP and pro- and anti-inflammatory cytokines were measured to assess immunological function.

## Materials and methods

### Test chemical and test diets

DEHP (D201154, purity ≥99.5%, Sigma-Aldrich, Zwijndrecht, The Netherlands) was dissolved in soy oil (Research Diet Services, Wijk bij Duurstede, The Netherlands), by gently stirring at room temperature for 2 min. Serial dilutions of this master solution and blank soy oil were mixed with the diet (NIH-07 diet, Research Diet Services, Wijk bij Duurstede, The Netherlands) before pelleting, aiming at concentrations in a range of 0.018–555.6 mg DEHP/kg feed, corresponding to targeted exposures of 0, 3.3, 33, 330, 3,300, 10,000, 33,000, and 100,000 μg/kg body weight/day (μkd) based on an average food consumption of 4.5 g/mouse (average body weight of 25 g)/day(d).

### Experimental conditions

This study was approved by the Animal Experimentation Ethical Committee of National Institute for Public Health and Environment under permit number 201100086 and carried out in accordance with prevailing legislation.

Nulliparous 13–14 weeks old female C57BL/6J mice (Charles River, Sulzfeld, Germany) were mated with 12–16 weeks old male FVB mice (GLP, Bilthoven, The Netherlands) to produce hybrid offspring with a known background information of phenotype and development (22). Mice were housed as previously reported (23). After an acclimatization period of 2 weeks, F0 female mice were fed experimental diets for 9 weeks starting 2 weeks pre-mating. Each dose group had six F0 females, which, to accommodate time and space restrictions, were mated in groups of three with one F0 male. Body weight and feed consumption of F0 females were monitored during pre-mating, gestation, and lactation. Anogenital distance of the offspring was assessed at post-natal day (PND) 4 and PND7 in the control, 3,300 and 100,000 μkd dose groups. Litter size was assessed at PND4 and PND21. At PND 21, after sacrifice by cervical dislocation under ketamine/xylazine anesthesia, serum of the dams was collected for DEHP metabolite determination.

From PND 21, offspring was weaned and housed individually (males-M) or in groups of 2–3 animals (females-F). Six litters of 5 or less pups (in dose groups 0, 33, 330, 10,000, 33,000 and 100,000 μkd) were discarded to avoid the effect of small litters on postnatal growth (23). After weaning, an average of 9 mice per sex (range 6–11) were included per dose group for follow-up through juvenile and adult stages. The control group consisted of 23 male and 19 female mice. Mice from available litters were randomly allocated to dose group using a computer-generated sequence, obtaining the following total number of individuals and litters, respectively per dose group/sex: 0 (M: 23/10; F:19/8), 3.3 (M:10/5; F:9/3), 33 (M:9/4; F:8/5), 330 (M:8/3; F:10/3), 3,300 (M:9/4; F:10/4), 10,000 (M:10/4; F:11/5), 33,000 (M:10/5;F:9/5) and 100,000 (M: 6/3; F:10/3). DEHP containing diet was discontinued at PND21 and offspring was further fed with the control NIH-07 diet. During the final 9 weeks of the study (starting at 46 weeks of age in males and at 48 weeks in females), all F1 offspring was challenged with a NIH-07 based HFD (D12451) containing 45 kcal% fat (lard) compared to 15 kcal% fat in the NIH-07 diet. Body weight was measured weekly from 5 to 55–57 weeks of age. In some experiments described below, a selection of control, middle (330 μkd) and/or high (33,000 μkd) DEHP dose groups was made to allow better allocation of resources and was based on evaluation of body weight changes such as for glucose homeostasis study or on the importance of observed parameter to middle or high exposures.

### *In vivo* experiments in adult F1 mice

Feed consumption was measured weekly in all F1 offspring at 21–23 weeks of age. Physical activity was measured in control and 33,000 μkd at 27–29 weeks of age. For this purpose, 10 animals per sex and per group were transferred to polysulfone cages mounted on Laboras platforms (Metris BV, Hoofddorp, The Netherlands). After an acclimatization period of at least 6 h, time spent in locomotion was continuously monitored for 36 h (males, individually caged) and 60 h (females, group caged) starting at 6.30 p.m. (begin dark phase) and expressed as kinetic energy indices per cage per 15 min as described in van Esterik et al. (23). At 30 and 31 weeks of age, a glucose tolerance test (GTT) with a 18-h fasting period and insulin tolerance test (ITT) were performed in both control and 33,000 μkd males and females offspring (24). Briefly, for the GTT a baseline glucose blood sample (0 min) in tail vein was taken before D-glucose injection i.p. at a concentration of 1.5 g/kg bw. At 15, 30, 60, and 120 min after injection, glucose was measured with a FreeStyle Lite meter (Abbott, Hoofddorp, The Netherlands). For ITT, glucose was measured before (0 min) and 15, 30, 45, and 60 min after insulin injection i.p. at a concentration of 0.75 IU/kg bw.

At 37–39 weeks of age, control and 33,000 μkd mice (*n* = 8–10 per sex per group) were subjected to an object recognition test (ORT) (24). Briefly, after habituation in the test cage, a training session (T1) started in which animals were exposed to two identical objects during 5 min. After a retention time of 120 min, test session (T2) took place by which animals were exposed to one familiar object and one novel object during 5 min. During both sessions, the time spent exploring each object was recorded with a stopwatch by the observer situated in front of the cage at 1 meter distance.

At 40 weeks of age, control and 33,000 μkd mice (*n* = 8–10 per sex per group) were subjected to a sucrose preference test as described previously (24). Briefly, animals were placed in a cage with two bottles filled with tap water and habituated for 4 days. Afterwards, a 4-day-test session was started in which a bottle filled with water and one with 1% w/v sucrose solution were available. Bottles were daily weighed and sucrose preference was calculated as percentage of sucrose water consumption out of total liquid consumption.

### Necropsy F1 mice

At termination of the *in vivo* study, after being fasted for 18 h to induce a general basal metabolic state, mice were sacrificed under ketamine/xylazine anesthesia. Nose-anus length, right femur length, body weight and glucose levels were assessed at dissection time. Liver, pancreas, spleen, brain, *m. quadriceps femoris*, thymus, adrenals, femur, testis, perigonadal fat, interscapular fat, and perirenal fat were weighed and fixed in formalin and/or liquid nitrogen. Formalin-fixed organs were stored at 4°C (except femur at room temperature), and after 24 h transferred to 70% alcohol. Blood samples were collected at the time of dissection by orbital puncture, treated with Pefabloc SC PLUS (Roche, Mannheim, Germany) to neutralize proteases, allowed to clot and centrifuged. Serum samples, snap-frozen organs and adipose tissue were stored at −80°C until further analysis. All F1 animals at this stage were fed a HFD so the measurements performed were under HFD condition.

### *Ex vivo* experiments

#### Internal dose measurement in F0

To avoid incorrect conclusions on internal exposure to DEHP due to contamination of samples with background levels of DEHP or its primary metabolite mono (2-ethylhexyl) phthalate (MEHP), secondary metabolites are preferred as biomarkers of DEHP exposure (8). Serum samples of 200 μl from dams on PND21 were analyzed for the presence of DEHP secondary metabolites: mono(2-ethyl-5-carboxypentyl)phthalate (MECPP), mono(2-ethyl-5-hydroxyhexyl) phthalate (MEHHP), and mono(2-ethyl-5-oxohexyl)phthalate (MEOHP) (9). Briefly, the analytical method includes an enzymatic deconjugation step, followed by solid phase extraction and quantitative analysis using isotope dilution. The chemical analysis was performed on an on-line trapping column in combination with liquid chromatography with mass spectrometric detection. In doses below 33 μkd, most metabolite concentrations were below the limit of quantification (LOQ), and for those samples, the equation LOQ/√2 was used to generate values for mean calculation (25).

#### Immune assessments in F1

Single-cell splenocyte suspensions were prepared from adult F1 mice controls, 330 and 33,000 μkd DEHP dose groups after exposure to a HFD by using fresh spleen as described in Tonk et al. (26). Splenocytes were plated 4 × 10^6^ cells/well in 24-well culture plates. Adherent splenocytes were stimulated with 15 μg/ml lipopolysaccharide (LPS) (Sigma) for 24 h and the supernatants were used to measure nitric oxide (NO) production using the Griess reaction, and tumor necrosis factor (TNF)-α production and IL-6 levels using an ELISA kit (eBioscience, San Diego, CA). Protein content was analyzed using the bicinchoninic acid method (Pierce Biochemicals, Rockford, IL) for cell number correction. Furthermore, splenocytes were seeded in a 96- well plate at 4 × 10^5^ cells/well and stimulated with 5 μg/ml concanavalin A (conA) or 15 μg/ml LPS for 48 and 24 h, respectively. Supernatants were used for the determination of interleukins: IL-1β, IL-2, IL-4, IL-10, IL-13 using a Milliplex Map kit (Millipore, Billerica, MA, USA) and interferon (IFN)-γ levels using an ELISA kit (eBioscience) as a measure of activation responsivity of these cells.

#### Serum chemistry in F1

Serum total cholesterol (CHOL), triglycerides (TGs), free fatty acids (FFAs), and high-density lipoproteins cholesterol (HDL-C) were analyzed as described (23). Adiponectin, leptin, ghrelin, insulin, glucagon, and C- reactive protein (CRP) were measured in sera by Milliplex Map Kit (Millipore) according to manufacturers' instructions. Glycated hemoglobin (HbA1c) was used as a marker for average glucose levels over the last 3 months (27), and assessed in full blood on a Beckman Coulter LX20 Clinical Chemistry Analyzer using the Direct Enzymatic HbA1c Assay kit (Diazyme Europe GmbH, Dresden, Germany). These measurements were performed after F1 animals switched to HFD.

#### Uncoupled protein 1 (*Ucp1*) gene expression analysis in F1

Gene expression of *Ucp1* was measured in the intrascapular fat tissue of controls and 100,000 μkd animals under HFD by qPCR as described previously (23). *Ucp1* is a marker of energy expenditure through thermogenesis, and contributes to regulation of body weight (28). In addition to *Ucp1*, expression levels of *Cidea* were determined as a marker of brown adipose tissue adipocytes (29) and were used to normalize the contents of brown adipose tissue adipocytes in the tissue extracts. Relative quantification was performed by the comparative CT method (ddCt).

### Statistical analyses

Data obtained from the whole range of doses tested, such as body weight measurements, endocrine, and lipid profile, were analyzed for statistically significant dose-response relationships using the benchmark dose (BMD) approach (30) with PROAST software (www.rivm.nl/proast), version 65.5. In this approach, models from exponential and Hill families were fitted to data covering the entire study population, and a BMD with its 5% lower (BMDL) and upper bounds (BMDU) of the 90% confidence interval was derived from the fitted models at a predefined benchmark response (CES = critical effect size) of 5%. The goodness of the fit was determined by Akaike information criterion (AIC). AIC integrates log-likelihood and the number of model parameters in one single value. The model with relatively low AIC gives a good fit without using too many parameters (31). The bootstrap method was used to calculate the 90% confidence interval of BMD so that individuals from the same litter were clustered to account for litter effects. Data which did not produce a statistically significant dose-response with exponential and Hill models as well as data with a wide confidence interval (BMDU/BMDL ratio>100) were considered not suitable for a valid BMD determination. Males and females were analyzed separately.

Measurements that included only a selection of dose groups such as neurobehavioral and immunological assessments were analyzed by a nested (to account for litter size covariance) ANOVA (using a custom R script), followed by Bonferroni *post-hoc* analysis (*p* < 0.025) to account for multiple testing (24). Males and females were analyzed separately.

## Results

### Internal exposure assessment

Analysis of the serum concentrations of MECPP, MEHHP, and MEOHP in dams following a 9-week dietary exposure to DEHP confirmed the presence of DEHP metabolites in the samples and, hence, internal exposure (Table 1). A positive correlation in serum concentrations of the secondary metabolites in relation to the nominal external DEHP doses was observed (*R*^2^ = 0.93 for MECPP, *R*^2^ = 0.99 for MEHHP, and *R*^2^ = 0.99 for MEOHP, Figure S1). Concentrations of secondary metabolite MEHHP and MEOHP in serum were in the same order of magnitude while the concentration of MECPP was the lowest (Table 1).

**Table 1:** Concentration of secondary metabolites MEOHP, MEHHP, and MECPP in serum of dams exposed via diet to DEHP.

**DEHP dose group (μkd)**	***N***	**MEOHP (ng/ml)**	**LOQ**	** < LOQ (%)**	**MEHHP (ng/ml)**	**LOQ**	** < LOQ (%)**	**MECPP (ng/ml)**	**LOQ**	** < LOQ (%)**
0	10	0.18 ± 0.06	0.17–0.41	100%	0.15 ± 0.04	0.14–0.34	80%	0.06 ± 0.03	0.04–0.10	40%
3.3	5	0.13 ± 0.01	0.18–0.22	100%	0.12 ± 0.02	0.15–0.18	20%	0.07 ± 0.04	0.04–0.05	NA
33	5	0.19 ± 0.10	0.18–0.43	100%	0.22 ± 0.06	0.15–0.35	60%	0.06 ± 0.02	0.04–0.11	60%
330	4	0.48 ± 0.16	0.17–0.30	25%	0.67 ± 0.40	0.13–0.25	25%	0.13 ± 0.05	0.04–0.08	25%
3300	6	3.18 ± 1.75	0.5–0.54	17%	5 ± 3	0.42–0.44	17%	1.05 ± 0.59	0.13	NA
10000	5	17 ± 3.16	0.3–0.53	NA	30.60 ± 10.06	0.25–0.43	NA	5.98 ± 2.38	0.08–0.13	NA
33000	6	27.92 ± 22.07	0.5–0.8	NA	54.04 ± 43.70	0.26–0.83	NA	8.05 ± 7.62	0.08–0.25	NA
100000	4	85.43 ± 56.03	1.7–1.9	NA	198.23 ± 131.83	1.4–1.6	NA	16.67 ± 14.75	0.43–0.48	NA

*N, number of samples; SD, standard deviation; LOQ, limit of quantification; < LOQ (%), percentage of the samples under the LOQ; NA, all samples above LOQ. Data shown as average± SD*.

### General toxicity and reproductive parameters

In dams, dietary exposure to DEHP had no effect on survival, behavior or body weight (Figure S2) Feed consumption was not affected by DEHP in the gestation weeks (3.9 ± 0.5 g/day) and in the lactation weeks (12.5 ± 5.5 g/day). Average reproduction rate was 87% (67–100% per dose group). The average litter size in the total of 45 litters was 7.0 (range 4–10), and litter sizes were evenly distributed over doses (Figure S3). No difference in the anogenital distance in F1 was observed (Figure S4). The overall F/M sex ratio in the F1 generation was 1.2 and the overall survival rate was 97%, with no effects of DEHP observed on these parameters.

### Energy balance

**Figure 1:**
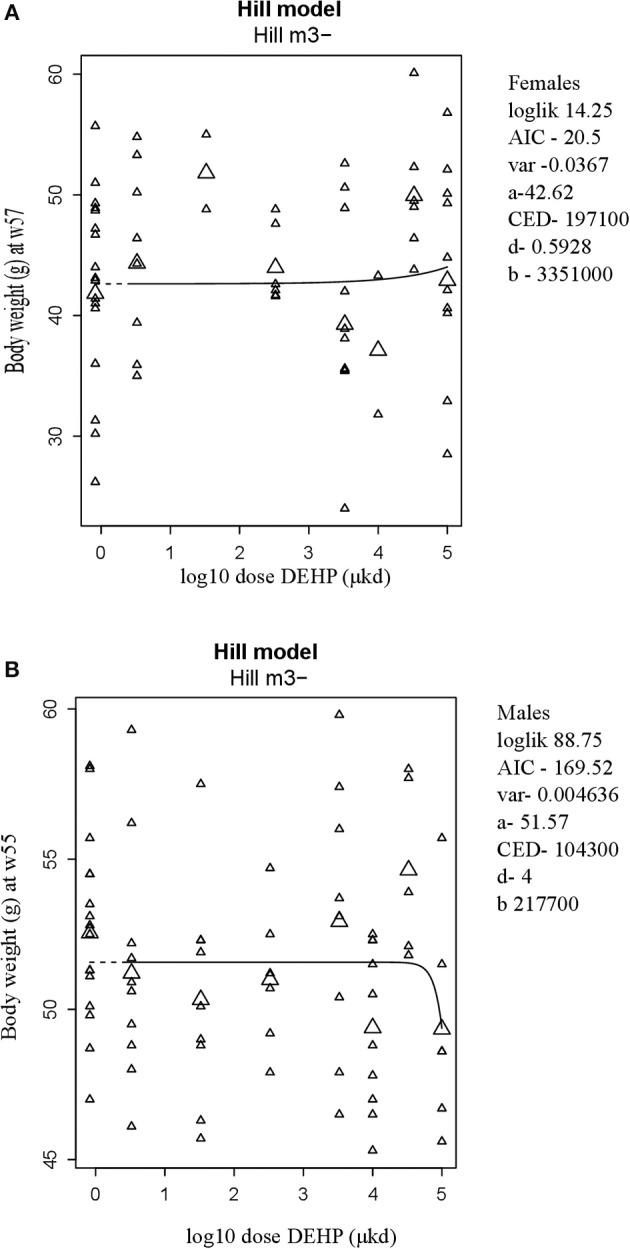
Body weight at 57 weeks of age in female **(A)** and at 55 weeks of age in male **(B)** offspring after developmental exposure to 3.3 to 100,000 DEHP μkd. Explanation of dose-response graphs is in Figure 2 legend.

A summary of dose-related effects on endpoints measured in F1 offspring exposed to DEHP during development is given in Table 2. No persistent changes in body weight were observed in F1 animals in either sex at the end of the follow-up period (55–57 weeks; Table 2, Figures 1A,B, Figure S5). In males, a dose-dependent decrease in body weight was observed until 4 weeks of age, but no effects on body weight were found after this period (Figures S6A,B). No effects on feed consumption were observed with the exception of a decrease in males at 23 weeks of age (data not shown), which did not coincide with a change in body weight. From 46 weeks of age until the end of the study (55–57 weeks), all animals were challenged with a HFD. No dose-dependent effects were observed in the body weight response during this period (Table 2). No difference in physical activity was observed between controls and 33,000 μkd exposed group in both sexes (Figure S7). No significant difference was observed in fat mass by individual analysis of perigonadal and perirenal fat weights in both sexes. In addition, no difference in *Ucp1* gene expression in intrascapular brown fat tissue between controls and exposed group 100,000 μkd was observed (data not shown).

**Table 2:** Overview of dose-response results in the offspring after *in utero* and lactational exposure to DEHP.

	**Males**				**Females**			
	**Dose response**	**BMDL μkd**	**BMDU μkd**	**Max effect size (%)**	**Dose response**	**BMDL μkd**	**BMDU μkd**	**Max effect size (%)**
**Body weight (bw)**								
Week 46 (m)/48 (f)	–				–			
Week 55 (m), 57(f)	–				–			
**Body size**								
Body length	–				–			
Femur length	–				–			
Femur length /bw	–				–			
**Growth**^a^								
Bw week 46/5 (m), 48/5 (f)	–				–			
Bw 55/46 (m), 57/48 (f)	–				–			
**Food consumption/bw**								
**Organ weights**								
Adrenal glands	–				–			
Brain	–				–			
Femur	–				↑	34,450	230,400	9
Liver	–				–			
***m.quadr.fem***.	↓	ni	ni	−8	–			
Pancreas	–				–			
Spleen	–				↑	8,350	24,800	22
Testes	–				–			
Thymus	–				–			
***Relative organ weights(/bw**^b^**)***	↓	ni	ni	−11	
***m.quadr.fem***
pancreas					↑	19,760	135,900	17
spleen						
**Fat pad weights**								
Interscapular	–				–			
Perigonadal	–				–			
Perirenal	–				–			
**Metabolic parameters**								
Cholesterol					–			
Free fatty acids	↑	2160	60,600	115	–			
High–density lipoproteins	↑	149	27,400	19	–			
Triglycerides	–				–			
Glucose(HbA1c)	↓	17,400	96,600	−13	–			
Glucose	–				–			
C–reactive protein	–				–			
Leptin	–				–			
Ghrelin	–				–			
Adiponectin	–				–			
Insulin	–				–			
Glucagon	–				–			

*↑,↓,- significant increase, decrease dose-responses, or absence of effect. Effects were significant with exponential (E) and Hill (H) modeling. BMDL and BMDU (lower and upper bounds of the 90%-confidence interval for BMD at a critical effect size of 5%); effects with a wide confidence interval of the BMD (ratio BMDU/BMDL> 100) are not considered informative (ni) for risk assessment. A maximum effect size is derived from the c-parameter if present in the dose-response function, otherwise calculated as a relative difference between top dose and control (background); reported value is an average of E and H maximum effect sizes*.

a *Growth is determined by the ratio bw in week 46 (males) or week 48 (females) to bw in week 5 and for the period in which animals are fed a HFD by the ratio bw in week 55 (males) or week 57 (females) to bw in week 46/48*.

b *Relative weight data are provided when significant dose-response in the parameter is observed*.

**Figure 2:**
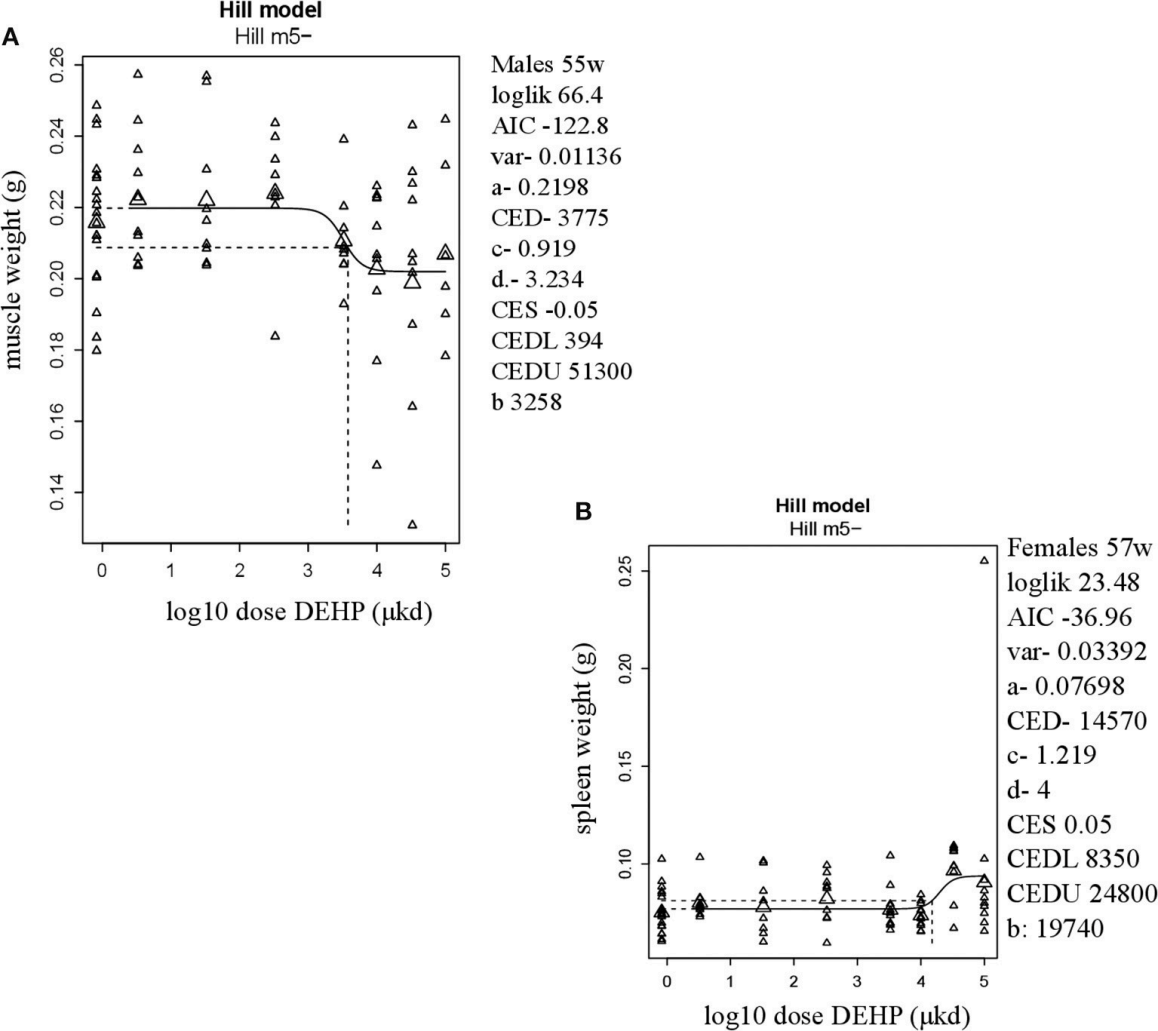
Dose-dependent changes in organ weights after 9 weeks of developmental exposure to DEHP. **(A)** decrease in muscle weight (*m.quadriceps femoris*) in 55 weeks of age male offspring; **(B)** increase in spleen weight in 57 weeks of age female offspring. The function of the curve is shown on the top of the chart. In the right corner version 65.5 of PROAST, parameters of significance of the fit [loglikelihood (loglik), AIC (Akaike information criterion) and variation (var)] together with the function parameters (a = background response, b = potency of chemical, c = maximum fold change in response compared to background response, and d = steepness of curve) that shape the curve are shown. CES, critical effect size. CEDL/CEDU, critical effect dose lower and upper bound of the (2-sided) 90%- confidence interval for the CED. Small triangles represent individuals and large triangles represent the geometric mean per dose.

In adult males, among all measured organs after necropsy, only muscle weight (*m.quadriceps femoris*) showed a dose-dependent decrease (Figure 2A). This 8% decrease persisted when expressed relative to body weight. However, a wide confidence interval (BMDU/BMDL ratio = 130) was observed and renders this parameter as not informative (Table 2). In adult females, a 9% increase in femur weight and a 22% increase in spleen weight were observed (Table 2). However, there was no effect in femur weight when analyzed relative to body weight, while the increase in spleen weight remained when expressed relative to body weight (Figure 2B), with a BMDL of 8,350 μkd.

**Figure 3:**
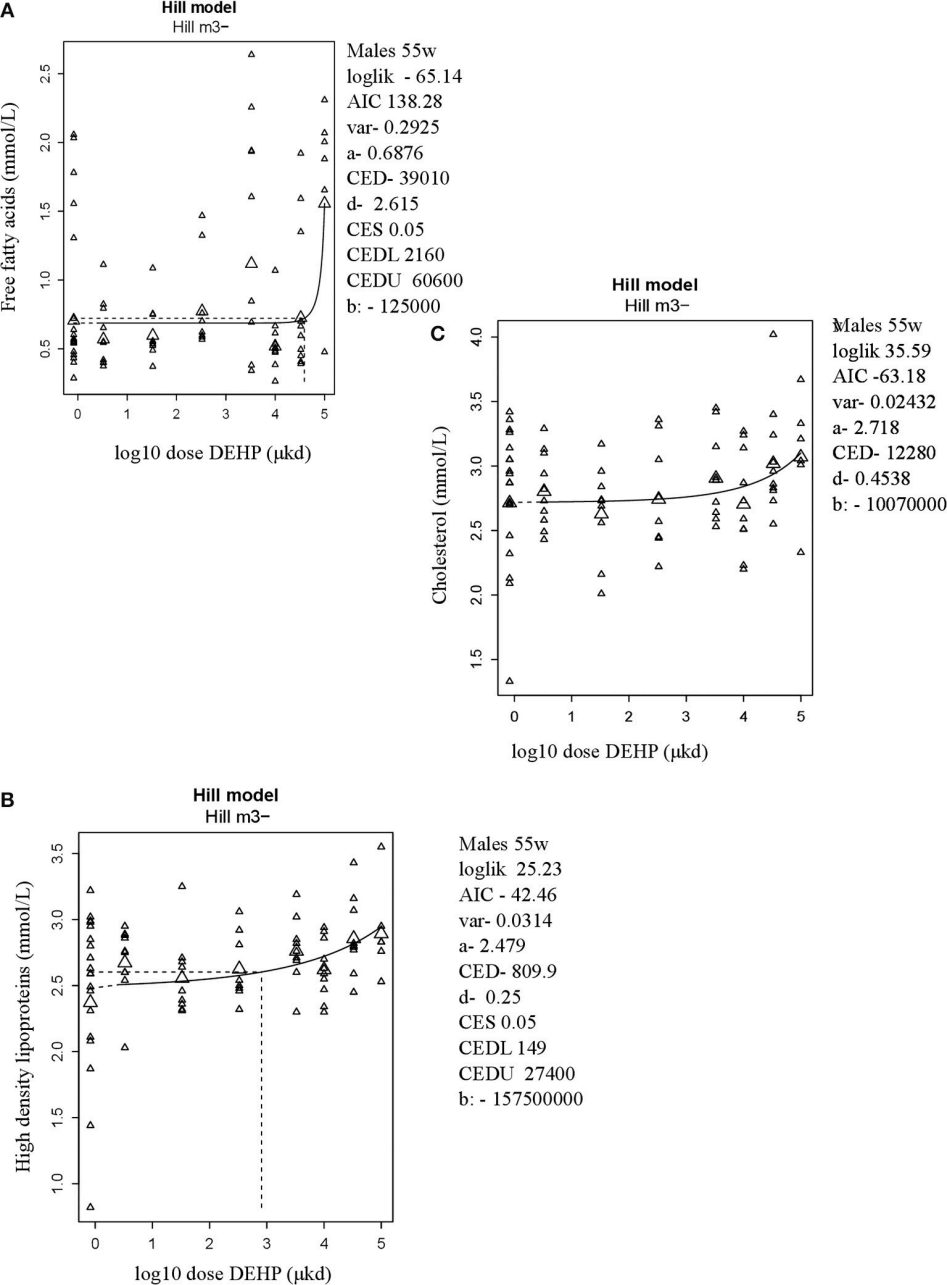
Dose-dependent increases in **(A)** Free fatty acids (FFA) and **(B)** High density lipoprotein (HDL-C) and no alteration in **(C)** Cholesterol in 55 weeks of age male offspring after developmental exposure to DEHP. Explanation of dose-response graphs is in Figure 2 legend.

In adult male offspring, dose-dependent increases in serum FFA and HDL-C (Figures 3A,B), but not TGs and CHOL (Figure 3C) were observed with FFA levels at the top dose 115% higher relative to background (Table 2). Considering the effect size and the BMDL of 2,160 μkd, the most critical parameter was the increase in FFA (Table 2). No effects on adiponectin, leptin, and ghrelin serum levels were observed. In females, no effects on serum lipids nor on endocrine parameters were observed (Table 2).

**Figure 4:**
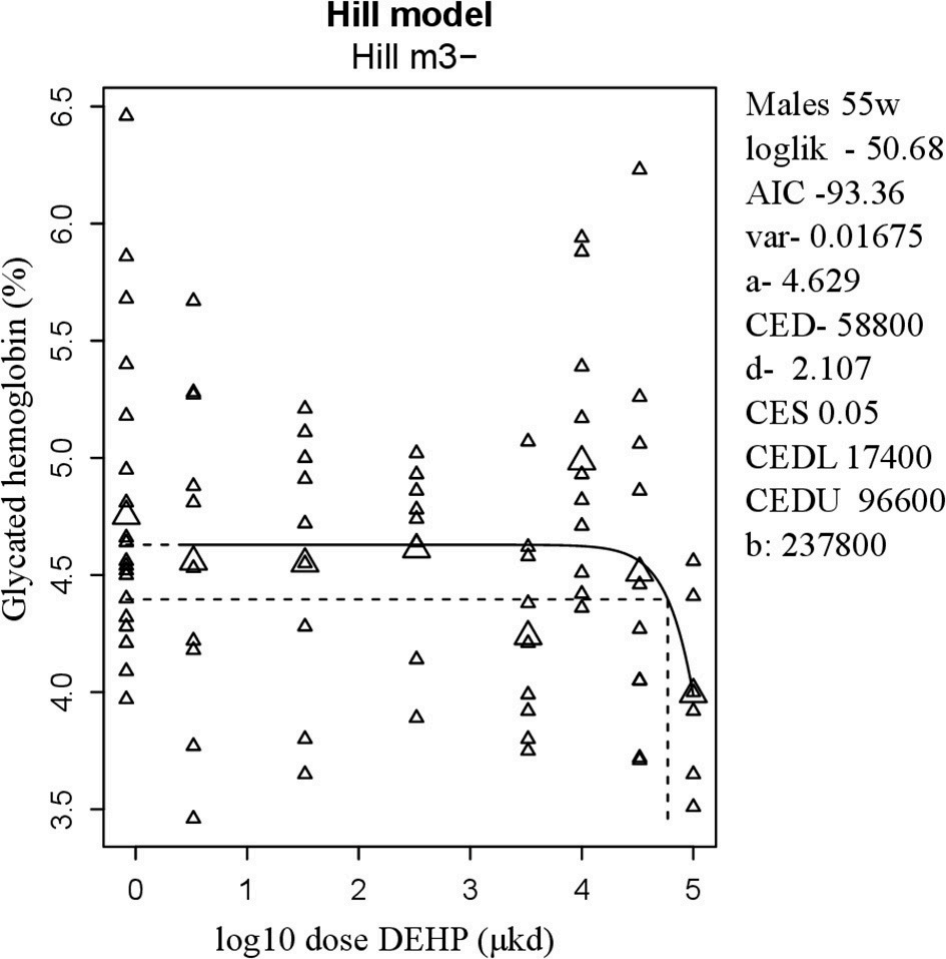
Dose-dependent decrease in glycated hemoglobin (HbA1c) in 55 weeks of age male after developmental exposure to DEHP. Explanation of dose-response graphs is in Figure 2 legend.

Glucose homeostasis appeared not to be affected by developmental DEHP exposure, as no difference between control and the 33,000 μkd exposed group was found in the GTT and ITT performed before start of HFD challenge. In addition, serum insulin and glucagon levels were not affected by exposure to DEHP when all dose groupswere analyzed. Fasting glucose levels measured during necropsy were also not altered. However, in males, HbA1c was decreased by 13% with a BMDL of 17,400 μkd (Figure 4).

### Neurobehavioral assessment

We examined the preference for a sucrose solution in adult F1 mice after developmental exposure to 33,000 μkd DEHP. No effect was observed compared to controls (data not shown).

**Figure 5:**
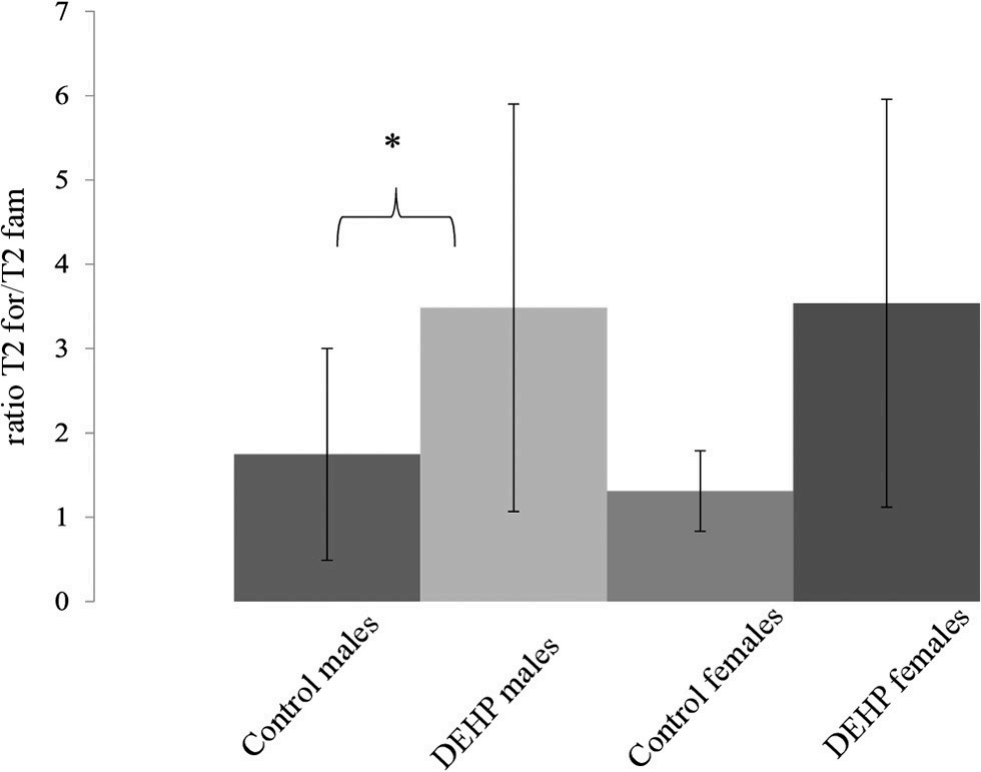
Effect of 33,000 DEHP μkd developmental exposure on the exploration performance during the object recognition test. The index Ratio T2for/T2fam is used and means the time exploring the foreign object (Tfor) divided by the time exploring the familiar object (Tfam) during the test session (T2). Columns represent average of the index calculated in males and females (controls × DEHP exposed with *n* = 8–10 animals/sex) and error bars depict standard deviation. Data was analyzed by a nested ANOVA, comparing exposed to control animals within sexes. ^*^p = < 0.05 (*p* = 0.0481, males; *p* = 0.0589, females).

In the ORT, total exploration time did not differ among control and 33,000 μkd exposed F1 mice within the sessions. During T2, exposed males showed a decrease in time exploring the familiar object (T2fam; Table 3) when compared to controls, which is reflected in a just significant (*p* = 0.0481) increase in the ratio between exploration time of the foreign object and the familiar object (ratio = T2for/T2fam), while in females the same trend but non-significant (*p* = 0.0589) was observed (Figure 5).

**Table 3:** Overview of exploration times and ratio during object recognition test (ORT).

**ORT**	**Males**		**Females**	
	**Control**	**DEHP**	**Control**	**DEHP**
Total T1 (s)	28.8 ± 11.6	27.6 ± 10.5	28.1 ± 13.6	18.8 ± 8.4
T2for (s)	11.5 ± 6.9	10.3 ± 4.1	17.1 ± 12.2	11.1 ± 9.2
T2fam (s)	7.8 ± 3.4	4.4 ± 3.3	13.4 ± 7.7	4.5 ± 3.7
Total T2 (s)	19 ± 7.8	15 ± 6.3	31 ± 19.1	16 ± 12.2
ratio T2for/T2fam	1.7 ± 1.3	3.5 ± 2.4^a^	1.3 ± 0.5	3.5 ± 2.4^a^

*Total T1: total exploration time during training session in seconds (sec); T2for: exploration time of foreign object during test session; T2fam: exploration time of familiar object during test session. Total T2: total exploration time during test session. Data shown as average ± SD. N (control males) = 10; N (DEHP males) = 10; N (control females) = 10; N (DEHP females) = 8*.

a *significance compared to controls within the same sex (p = 0.0481, males; p = 0.0589, females)*.

### Immunological assessment

**Figure 6:**
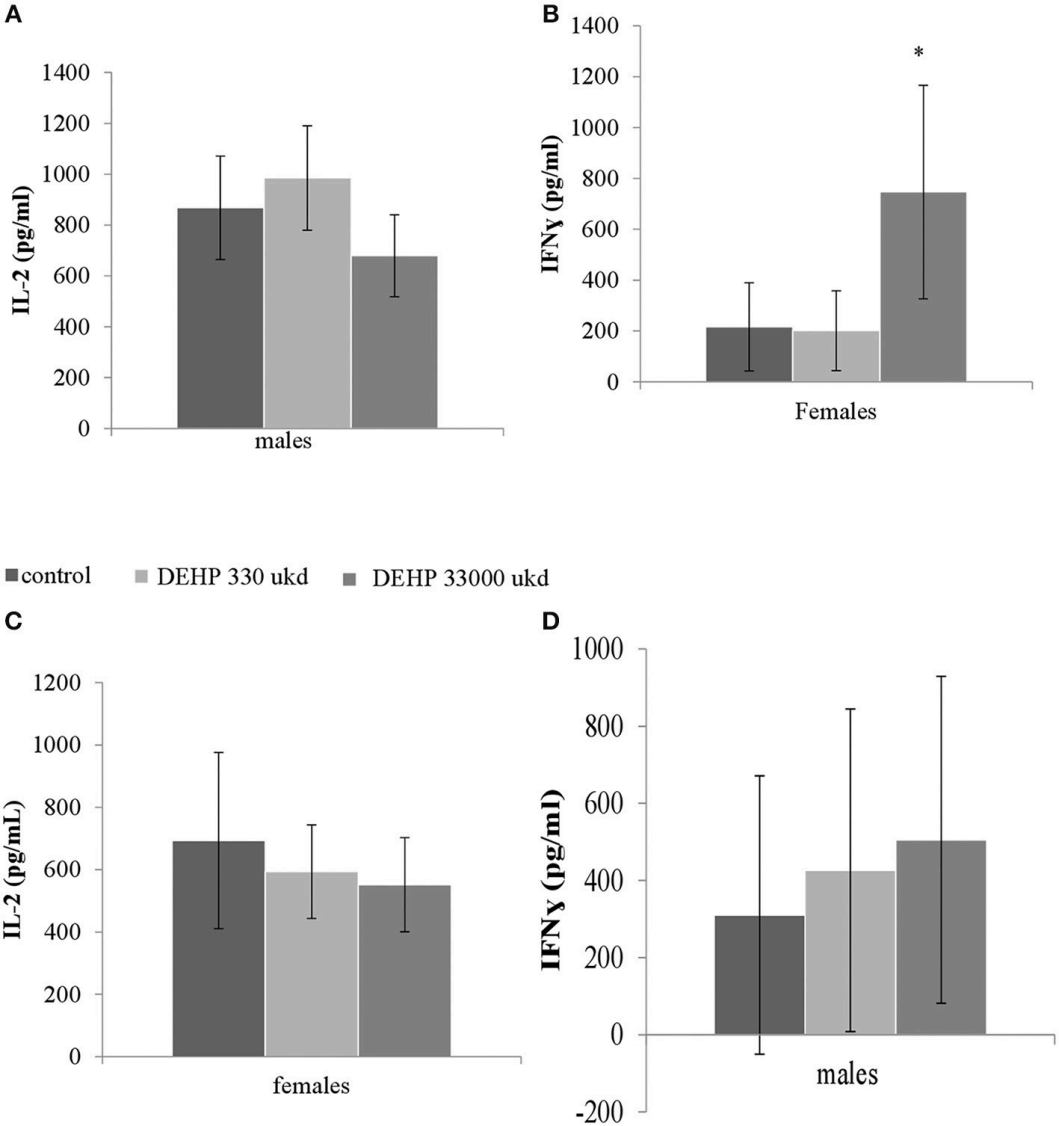
Sex-specific effects of developmental exposure to DEHP on immunological parameters measured *ex vivo* in splenocytes culture after ConA stimulation. No effect on IL-2 in males **(A)** and a significant effect on IFNγ in females (^*^ means *p* = 0.01) **(B)**. No effects on IL-2 in females **(C)** nor on IFNγ in males **(D)**. Columns represent average of concentrations measured in males and females (controls × DEHP exposed with *n* = 8–10 animals/sex) and error bars depict standard deviation. Data was analyzed by a nested ANOVA followed by a *post-hoc* analysis (*p* < 0.025).

Serum levels of CRP were not affected in adult offspring exposed to DEHP during development (Table 2). No significant differences were observed in NO, IL-6 (Figures S8A,B) and TNFα production in adherent splenocytes after *ex vivo* stimulation with LPS in both sexes after developmental exposure to DEHP at 330 and 33,000 μkd. Concerning cytokine production after ConA stimulation, *post-hoc* analysis showed a non-significant decrease in IL-2 levels in F1 males toward the 33,000 μkd DEHP exposed group (*p* < 0.08, Figure 6A). In F1 females, a significant increase in IFNγ (*p* < 0.01) was observed at 33,000 μkd DEHP exposed group (Figure 6B). No other cytokine was affected by the exposure to DEHP during development as shown in the examples for IL-2 in females and IFNγ in males (Figures 6C,D).

## Discussion

In this study we investigated the metabolism disrupting properties of developmental exposure to DEHP in combination with effects on neurobehavior and immunological functions. We mimicked human dietary exposure to DEHP by including low doses to our range of studied concentrations. We observed alterations in lipid metabolism, glucometabolism, and neurobehavior in adult C57BL/6JxFVB hybrid male mice, as well as in immune function in adult female mice.

Initially, we measured the internal concentrations of DEHP secondary metabolites MEOHP, MEHHP, and MECPP in serum from dams. An increased concentration of all metabolites highly correlated with the external dose, confirming internal exposure. We show here that in the serum from DEHP exposed dams, MEHHP, and MEOHP are present in the highest concentrations, followed by MECPP. This is in line with previous studies examining concentrations of DEHP metabolites in mouse urine (32). Limited studies have measured DEHP in human serum samples, in either cord blood (9) or adult plasma (33). Levels of these three metabolites at our external DEHP exposure of 330 μkd were similar to background levels observed in human cord blood [0.29–0.31 ng/ml; (9)]. In contrast to data in rodents, likely due to inter-species metabolic capacity and pathways (32), concentrations of MEOHP, MEHHP, and MECPP in humans are similar in cord blood, whereas in adult blood, higher levels of MECPP are found relative to the other secondary metabolites. In a recent study, Quinnies et al. (34) reported serum levels of MEOHP in dams exposed to 40 and 400 μkd DEHP. MEOHP values of 0.54 ng/ml for the lowest dose and 1.6 ± 0.4 ng/ml for the highest dose were within the range of our exposures of 330 μkd and 3,300 μkd, respectively. They also report similar levels of MEOHP for the dose of 400 μkd in embryos, confirming that secondary metabolites reach the offspring. The primary DEHP metabolite MEHP is considered the active metabolite of DEHP, but Engel et al. (35) reported that the secondary metabolites are PPARα and/or PPARγ agonists *in vitro*, indicating that also the secondary metabolites may be involved in the molecular mechanisms behind the DEHP effects. Although the metabolites all have short half-lives ranging from 5 to 15 h (8), exposure at a developmental stage is likely to be implicated in the persistent effects of DEHP as discussed below.

During the first 26 weeks of life, we mainly focused on exploring changes in body weight and feed consumption in the offspring. We observed transient increases in body weight in females and transient decreases in body weight in males in a few weeks, but taking the entire follow up period into account, no clear impact on body weight was observed. In line with our findings, recent studies report a lack of effects on body weight after developmental exposure to DEHP (34, 36, 37). After 27 weeks of age, we continued with the body weight measurements, performed a locomotion test and studied the glucose homeostasis via glucose and insulin tolerance tests. With no further changes in body weight nor fat mass, no alteration in physical activity and normal glucose and insulin levels, we did not detect any changes which could be an indication of metabolic disruption through adipogenesis or endocrine pancreas function by developmental exposure to DEHP. Our findings do not support the results of a recent meta-analysis (14) which showed that early life exposure to DEHP is significantly associated with increased fat weight, but not body weight. It should be noted, however, that the authors judged the quality of evidence for body weight and fat weight to be low due to concerns regarding risk of bias and unexplained inconsistency (i.e., substantial heterogeneity).

We performed a sweet preference test and an object recognition test to check whether early exposure to DEHP could promote changes in neurobehavior between 37 and 40 weeks of life. Using these tests, we report that male offspring had changes affecting their attention span, particularly toward the familiar object. Although these changes were not accompanied by an increased preference for a sweet beverage, it is an indication of impaired neurobehavior in line with Barakat et al. (17). Barakat reported elevated anxiety and impaired memory function in male mice exposed early in life to DEHP as signs of developmental defects in the neural system or neurodegeneration caused by inflammation and/or oxidative damage in the hippocampus. More research is needed to investigate the mechanisms underlying such neurobehavioral changes and the impact of those changes in the context of MetS.

To further study the effects of developmental exposure to DEHP, we measured lipids levels in serum of 55–57 weeks of age mice and report a dose-related increase in free fatty acids and high density lipoprotein cholesterol in our male offspring. In lipid metabolism, triglycerides stored in adipose tissue are hydrolyzed to glycerol and free fatty acids. When lipolysis is stimulated, an increase in free fatty acids is expected. As fatty acids are the precursors of cholesterol, an increase in cholesterol may occur. To cope with cholesterol increase, HDL may be mobilized to transport cholesterol to the liver and facilitate removal (38). Gu et al. (36) reported that after gestational exposure to 50 μkd to DEHP via gavage, 9 weeks old male and females had an increase in visceral fat weights associated with elevated levels of triglycerides and total cholesterol. Hao et al. (11) also showed that maternal exposure from gestational day 12 to PND 7 to 250 μkd DEHP via gavage results in elevated cholesterol and triglycerides at 8 weeks of age while maternal exposure to 30,000 μkd DEHP by gavage from 4 weeks prior to gestation to PND 28 resulted in a significant increase in serum cholesterol, but not triglycerides, in offspring at 8 weeks of age (39). The studies above report alteration in serum lipids under a normal diet. In our experimental setting, we studied for the first time the effect of developmental exposure to DEHP in combination with a HFD challenge as calorie-rich diets are tightly linked to the pandemic of metabolic disorders (37). We pinpoint here increases in 2 parameters out of 4 related to lipid metabolism studied (TG's, FFA, CHOL, HDL-C). Although these should be interpreted with caution in view of limited effect sizes, our findings strengthen the body of evidence that developmental exposure to DEHP disrupts lipid metabolism.

Experimental studies indicate that DEHP targets lipid and cholesterol metabolism. *In vitro* and *in vivo* studies suggest that cholesterol transport into mitochondria needed for steroid biosynthesis is inhibited by DEHP, leading to accumulation of lipid droplets, while *de novo* synthesis of cholesterol is stimulated by DEHP (40). Although our offspring was indirectly exposed to DEHP via maternal diet, it is likely that direct effects of DEHP on somatic cells of the developing embryo/fetus may affect proliferation, differentiation and organ development. Recent data suggest that the adrenals are specific target organs of developmental DEHP exposure that may play an important role in the metabolic effects of DEHP, as global gene expression study has revealed changes in lipid metabolism and PPAR pathways affected in adult adrenal glands after *in utero* exposure in a rat model (41). In addition, another developmental *in vivo* study in mice related increased serum cholesterol levels in the offspring to decreased hepatic clearance of cholesterol, as suggested by decreased protein expression of cholesterol clearance-related regulators (39). These reports point to adrenals and liver as target organs and give insight into the long term effects of DEHP exposure on lipid metabolism, but the mechanism is still not fully understood. In the case of adrenals as a target organ of DEHP, involvement of epigenetic regulation remains to be elucidated as the differential DNA methylation identified did not affect directly gene expression (42).

In addition to effects on lipid metabolism, our study indicated that glucose homeostasis may be affected by developmental DEHP exposure. Serum levels of glycated hemoglobin, a marker of long term glucose levels, were decreased in adult male offspring. In previous studies, it has been shown that developmental exposure of rodents to DEHP alone or in combination with a high-fat dietary challenge disrupts glucose homeostasis in offspring (13, 43, 44). In these studies, disruption of glucose homeostasis was accompanied by effects on glucose and insulin tolerance. In our study, we performed GTT and ITT in one treatment group only (33,000 μkd) and observed no effects. Non-monotonic dose-response relationships have been reported for several EDCs, making it difficult to predict effects at lower doses by using higher doses (45). However, as all other endpoints measured in this study to investigate glucose regulation at 55–57 weeks of age (i.e., serum levels of fasting glucose, insulin and glucagon levels and glycated hemoglobin) did not show non-monotonic dose-responses, lower dose effects on GTT ad ITT are not expected. Overall, as our finding of decreased glycated hemoglobin is limited to alteration in one single parameter and is in opposite direction of the effects mostly associated with insulin resistance, it should be interpreted with caution.

We also measured a range of immune parameters in the cell population present in spleen after stimulation and observed sex-specific effects on cytokines, but not on CRP, a marker of chronic low grade systemic inflammation. Following developmental exposure to 33,000 μkd, splenocytes isolated from adult females produced an increase in IFNγ after stimulation with Con A, which is a T-cell response. Female offspring also showed a dose-related decrease in spleen weight, with a BMDL of 9,189 μkd, suggesting that developmental exposure to DEHP may affect immune functions in female offspring. Low grade inflammation plays a role in the development of obesity and metabolic disorders and in a recent report (19), elevated serum levels of CRP and TNF-α in 300,000 μkd DEHP *in utero* exposed offspring were detected. IFNγ is a pro-inflammatory cytokine and contributes to metabolic dysfunction by the repression of the expression and activity of SIRT1, an energy sensor, resulting in altered expression of genes involved in cellular metabolism and energy expenditure (46). In addition, patients with type 2 diabetes show elevated IFNγ circulating levels (46). However, it cannot be excluded that an elevated IFNγ represents a non-adverse physiological response to DEHP in combination with ConA exposure (47). In contrast to our findings, in another study with younger animals with follow-up until 13 weeks of age, *in utero* exposure of CD female rats from GD 6-12 by gavage to DEHP at doses of 37,500, 75,000, 150,000, or 300,000 μkd DEHP showed no effects on immune organ weights, antibody levels and *ex vivo* cytokine production (48). Taken together, the evidence in animal studies on the effects of developmental exposure to DEHP on the immune system in the context of metabolic disorders is limited and more research is needed.

The strength of our study is the wide range of doses studied, from 3.3 to 100,000 μkd DEHP. The dose regime applied also mimicked the relevant human exposure route, i.e., through the diet, and included the important periods of development encompassing both gestation and lactation. The lower dose ranges applied approximated human external exposure concentrations, which have been estimated to range from 2.5 to 15.7 μkd for an average adult of 60 kg, and about 24 μkd for an infant in the first year of life (20). The highest dose used is slightly above NOAEL levels, i.e., 91,000 μkd reported for a two generation developmental toxicity study in CD-1 mice (21). The results observed in our study are exclusively at the higher external dose range and the most critical BMDL of 2,160 μkd for FFA, if used to calculate a margin of safety (MOS), is >100 times higher than an average human exposure (49), though it should be noted that the internal serum concentrations of DEHP secondary metabolites in dams at this BMDL approximated those reported human cord blood (9). We did not observe developmental toxicity in terms of anogenital distance in offspring or otherwise, but we did observe a transient decrease in the body weight of the dams during the first weeks of gestation. To rule out possible toxic effects of the top dose (100,000 μkd) tested, we excluded this dose group from subsequent analyses of glucose homeostasis, immune function, and neurobehavior. In addition, we had a year follow-up, with parameters measured during the whole study period, so we could investigate the development of adult disease following the initial early exposure to DEHP.

We observed sex-specific effects of DEHP on lipid metabolism, neurobehavior, and immune function, though the mechanisms underlying these effects warrant further study. The sex-specific anti-androgenic effects of DEHP on male sexual development and reproduction are well known (50, 51) and sexual dimorphic expression of genes controlling hepatic lipid metabolism could play a role in the different outcome between sexes as, in an obesity context, transcription factors/nuclear receptors response to pollutants is sex-dependent (52). There is also evidence that sex influences innate and adaptive immune responses. Sex chromosome genes and sex hormones, including estrogens, progesterone, and androgens, contribute to the differential regulation of immune responses between the sexes (53). For instance, half of the activated genes in female T cells have estrogen response elements (ERE) in their promoters, suggesting that sex steroids may directly cause dimorphic immune responses (54). Therefore, it is expected that environmental factors such as DEHP exposure may alter the development and function of the immune system differently in males and females.

Our research suggests that developmental exposure to DEHP is a long term dyslipidemic factor, due to the observed changes in total cholesterol, FFA and HDL-C reported at adulthood. Dyslipidemia is a leading cardiovascular risk factor characterized by high circulating triglycerides, total cholesterol and LDL-C and low HDL-C; it affects 20% of children and adolescents in the U.S. (55) and is associated with future cardiovascular and metabolic disease risk (56). Therefore, developmental exposure to DEHP should be further investigated to understand potential risks to human health. Our most critical parameter was the circulating FFA levels. Although an increase in FFA cannot be claimed as adverse, it is suggested that FFA can mediate adverse metabolic effects such as insulin resistance (57). Developmental exposure to DEHP may either directly activate fatty acid catabolism (58) or indirectly via changes in expression of genes related to beta-oxidation (58), leading to increased levels of FFA at adulthood observed here. FFA are also important signaling molecules and an increase may affect brain and endocrine pancreas functions (15). Therefore, the neurobehavior and lipid metabolism effects detected in our study could be linked with one another by the circulating FFA.

In conclusion, developmental exposure to DEHP *in utero* and during lactation resulted in modest metabolic changes in lipids and glucose in combination with a neurobehavioral change in the area of attention span in adult C57BL/6JxFVB male mice at 55 weeks of age. The most critical and sensitive alteration was on FFA in serum which warrants further investigation on adversity.

## Author contributions

JL, LvdV, and TH supervised the project. LvdV, JL, JvE, and LB designed the experiments. JvE and LB carried out the experiments. HH contributed to sample preparation. ML and TH assisted with metabolites analysis. LB wrote the manuscript with input of all authors.

### Conflict of interest statement

The authors declare that the research was conducted in the absence of any commercial or financial relationships that could be construed as a potential conflict of interest.

## Abbreviations

BMD: Benchmark dose
BMDL/BMDU: lower/upper bound of the 90%-confidence interval for BMD at a critical effect size of 5%
CHOL: cholesterol
conA: concanavalin A
CRP: C-reactive protein
DEHP: di (2-ethylhexyl) phthalate
DOHaD: developmental origins of health and disease
FFA: free fatty acids
GTT: glucose tolerance test
HbA1c: Glycated hemoglobin
HDL-C: high density lipoprotein cholesterol
HFD: high fat diet
IFN-γ: interferon γ
ITT: insulin tolerance test
LOQ: limit of quantification
LPS: lipopolysaccharide
MECPP: mono(2-ethyl-5-carboxypentyl)phthalate
MEHHP: mono(2-ethyl-5-hydroxyhexyl) phthalate
MEHP: mono (2-ethylhexyl) phthalate
MEOHP: mono(2-ethyl-5-oxohexyl)phthalate
MetS: metabolic syndrome
NOAEL: no observed adverse effect level
NO: nitric oxide
PND: postnatal day
ORT: object recognition test
TGs: triglycerides
TNF: tumor necrosis factor
T1: training session
T2: test session
μkd: μg/kg body weight/day.
