# Supplementary material for: Analysis of Lipid Metabolism, Immune Function, and Neurobehavior in Adult C57BL/6JxFVB Mice After Developmental Exposure to di (2-ethylhexyl) Phthalate
Source: Front Endocrinol (Lausanne). 2018 Nov 21;9:684. doi: 10.3389/fendo.2018.00684 (PMC6259287; doi:10.3389/fendo.2018.00684)

Supplemental material

**Title:** Developmental exposure to di (2-ethylhexyl) phthalate affects lipid metabolism and neurobehavior in adult C57BL/6JxFVB male mice offspring

**Authors:** Liana Bastos Sales^1^, Joantine CJ van Esterik ^2^, Hennie M Hodemaekers^3^, Marja H Lamoree^1^, Timo HM Hamers^1^, Leo TM van der Ven^3^, Juliette Legler^4,5*^

^1^Department of Environment and Health, Vrije Universiteit Amsterdam, The Netherlands

^2^ Department of Pathobiology, Faculty of Veterinary Medicine, Utrecht University, Utrecht, The Netherlands

^3^ Center for Health Protection, National Institute for Public Health and the Environment (RIVM), Bilthoven, The Netherlands

^4^ Institute for Risk Assessment Sciences (IRAS), Utrecht University, Utrecht, The Netherlands

^5^ Institute for Pharmaceutical Sciences, Utrecht University, Utrecht, The Netherlands

*** Correspondence:** Juliette Legler (j.legler@uu.nl)

Figure S1 – Positive correlation between nominal external DEHP doses given via diet to dams and average of metabolites concentrations measured in serum of females at PND21.

Figure S2 – No alteration in body weight in dams after 6 weeks of exposure to DEHP via diet. The function of the curve is shown on the top of the chart. In the right corner, parameters of significance of the fit (loglikelihood (loglik), AIC (Akaike information criterion) and variation (var) together with the function parameters (a=background response, b= potency of chemical, c= maximum fold change in response compared to background response and d=steepness of curve) that shape the curve are shown. CES=critical effect size. Small triangles represent individuals and large triangles represent the geometric mean per dose. Analysis was performed with PROAST version 65.5.


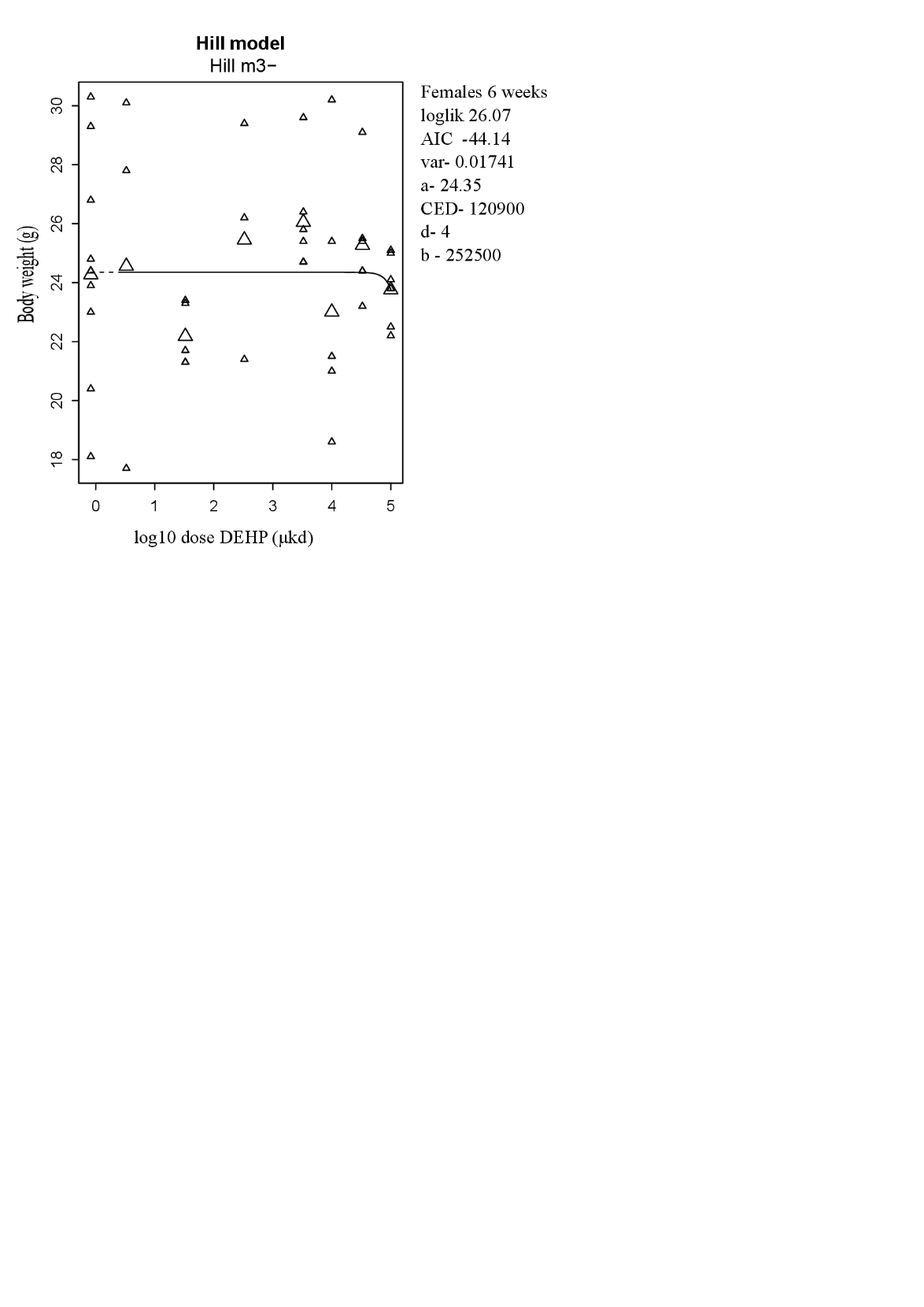


Figure S3 – No alteration in litter size after developmental exposure to DEHP. Explanation of the dose-response graph is in Fig. S2 legend. Triangles next to litter size n=2 represent females with no litters.


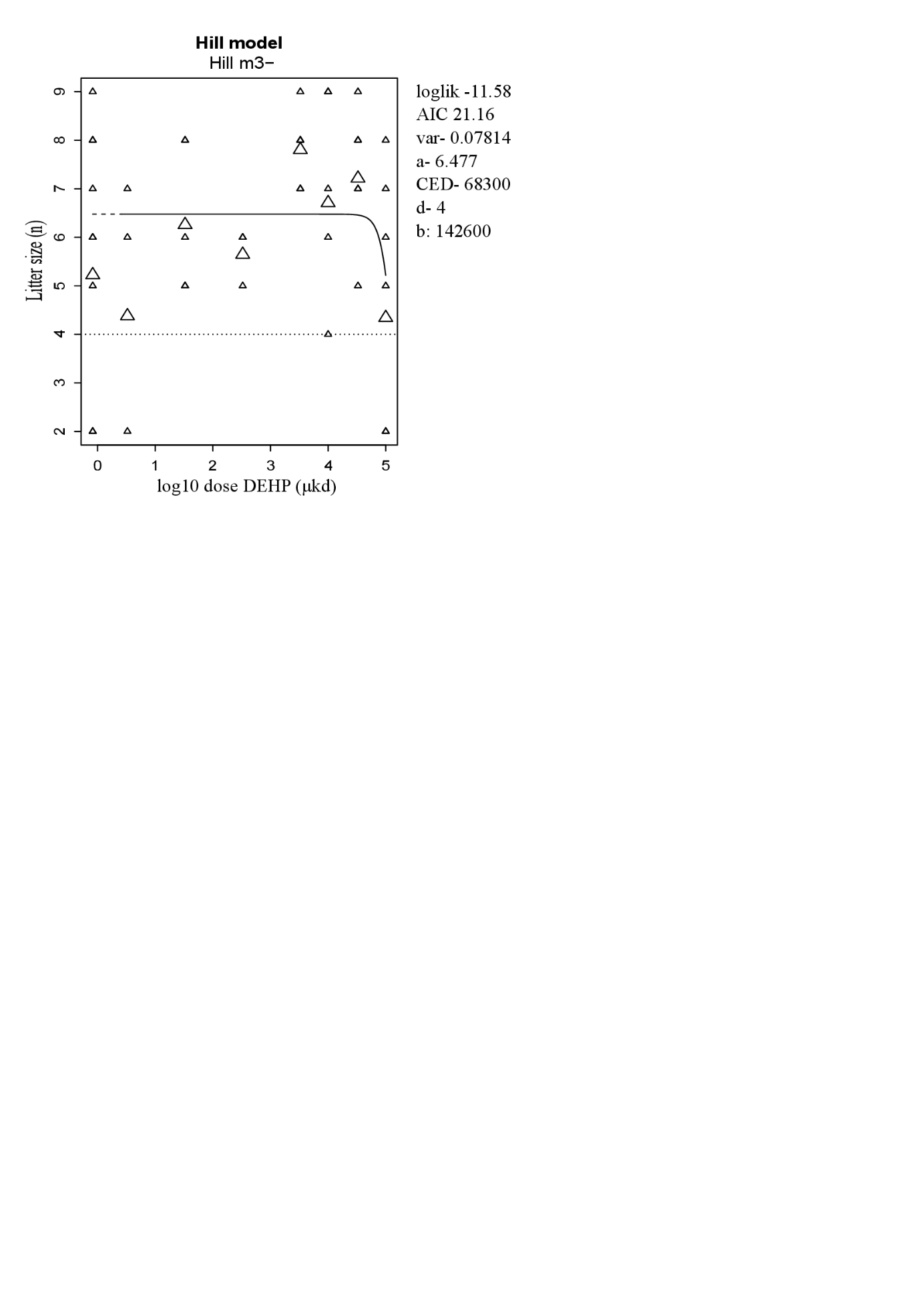


Figure S4- No effect of developmental DEHP exposure on anogenital distance related to body weight measured at postnatal day (PND) 4 and 7. N=29, n=16 and n=9 for males and N= 29, n=31 and n=17 for females at 0, 3300 and 100000 µkd, respectively.

Figure S5 – No body weight changes per dose group across the follow-up period. (A) Males from 1 to 55 weeks of age and (B) Females from 1 to 57 weeks of age.

(A)

(B)

Figure S6- Decrease in body weight in male offspring at 4 weeks of age (A) and no effects after that period as at 8 weeks of age (B). Explanation of the dose-response graph is in Fig. S2 legend.


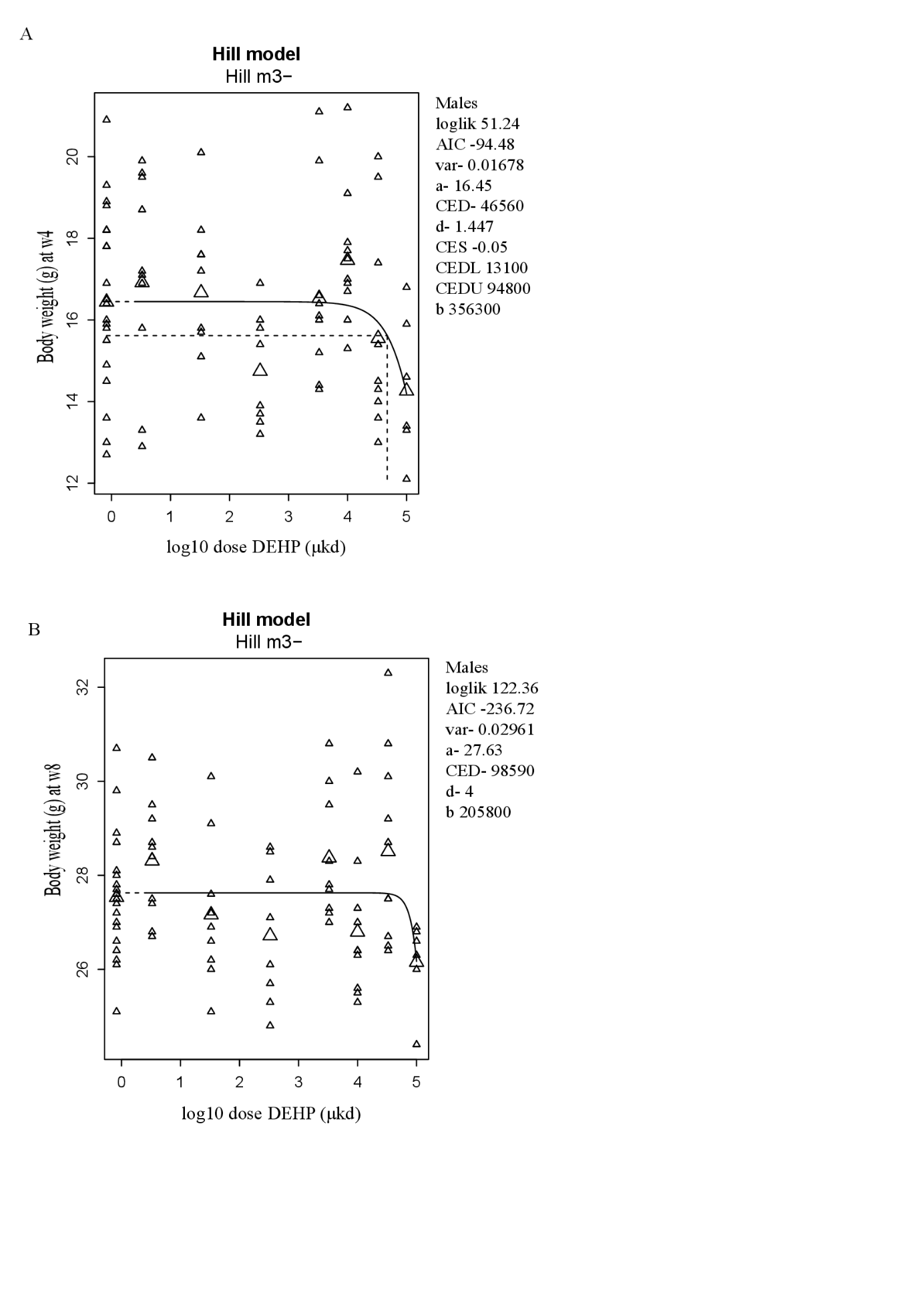


FigS7. No effect of developmental exposure to DEHP on physical activity. (A) Females during 60 hours of monitoring and (B) Males during 36 hours of monitoring. Physical activity is defined as time spent in locomotion and expressed as kinetic energy indices per cage per 15 min.

(A)

(B)

Fig S8. No significant differences in IL-6 levels in male (A) and female (B) offspring after developmental exposure to DEHP at 330 and 33000 µkd in adherent splenocytes after *ex vivo* stimulation with LPS.

A


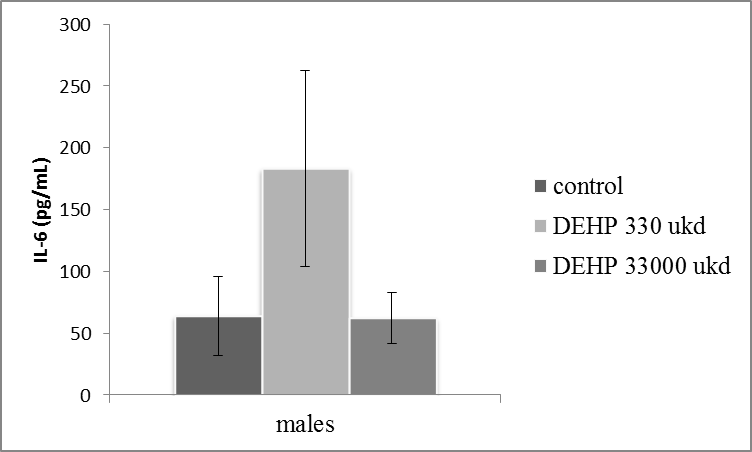


B


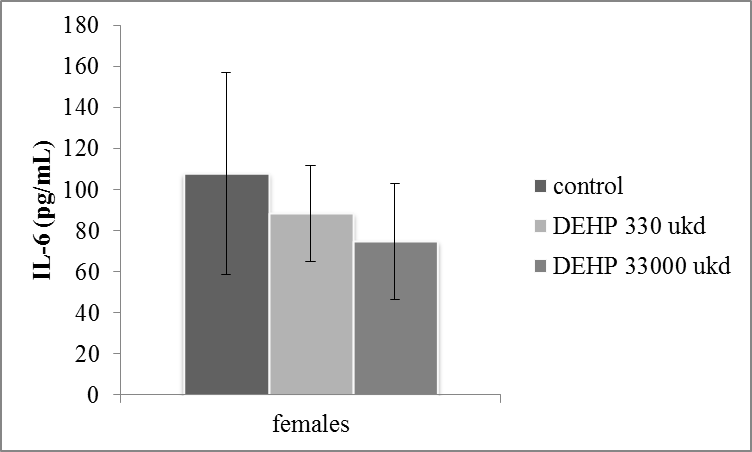

Supplement: Supplementary file 1 [file Data_Sheet_1.docx]
